# A humanized yeast-based toolkit for monitoring phosphatidylinositol 3-kinase activity at both single cell and population levels

**DOI:** 10.15698/mic2018.12.660

**Published:** 2018-11-12

**Authors:** Julia María Coronas-Serna, Teresa Fernández-Acero, María Molina, Víctor J. Cid

**Affiliations:** 1 Departamento de Microbiología y Parasitología, Facultad de Farmacia. Universidad Complutense de Madrid e Instituto Ramón y Cajal de Investigaciones Sanitarias (IRYCIS).

**Keywords:** PI3K, p110α, phosphoinositides, Saccharomyces cerevisiae, humanized yeast, heterologous expression, fluorescent reporter, eisosomes, septins, kinase inhibitors

## Abstract

Phosphatidylinositol 3-kinase (PI3K) is a key regulator of phosphoinositide-dependent signaling in mammalian cells and its dysfunction is related to multiple syndromes, including cancer. By heterologous expression in *Saccharomyces cerevisiae*, we have developed a humanized yeast system as a tool for functional studies on higher eukaryotic PI3K. Here we restrict PI3K activity in yeast to specific plasma membrane (PM) microdomains by fusing the p110α PI3K catalytic subunit to either a septin or an eisosome component. We engineered a Dual Reporter for PI3K (DRAPIK), useful to monitor activity on cellular membranes *in vivo* at a single-cell level, by simultaneous PM staining of the enzyme substrate (PtdIns4,5P_2_) with GFP and its product (PtdIns3,4,5P_3_) with mCherry. We also developed a sensitive FLUorescence by PI3K Inhibition (FLUPI) assay based on a GFP transcriptional reporter that is turned off by PI3K activity. This reporter system proved useful to monitor PI3K inhibition *in vivo* by active compounds. Such novel tools were used to study the performance of yeast PM microdomain-directed PI3K. Our results show that tethering heterologous PI3K to discrete PM domains potentiates its activity on PtdIns4,5P_2_ but different locations display distinct effects on yeast growth and endocytosis.

## INTRODUCTION

Class I phosphatidylinositol 3-kinases (PI3Ks) are responsible for conversion of PtdIns4,5P_2_ into PtdIns3,4,5P_3_ in eukaryotic cells. Inhibitors of this enzymatic activity have thoroughly been sought, as its product is a second messenger involved in proliferative, anti-apoptotic and inflammatory pathways, among others [1]. By heterologous expression in the *Saccharomyces cerevisiae* eukaryotic unicellular model, which naturally lacks such PI3K activity, we have previously engineered a system to *in vivo* titrate the activity of pathologic mutant versions of components of the human PI3K pathway, namely the p110α catalytic [2-4] and p85 regulatory subunits [5] that conform the class IA PI3K, its main effector Akt [4, 6] and its key down-regulator, the tumor suppressor PTEN [7, 8]. In order to meet its substrate, the PI3K p110α catalytic subunit must be directed to the plasma membrane (PM). In mammalian cells, spatial regulation occurs by recruitment via the p85 regulatory subunit, which recognizes phospho-Tyr residues generated by local ligand-mediated activation of tyrosine kinasecoupled receptors [9, 10]. The PI3K effector Akt1 is subsequently anchored to the PM via its PtdIns3,4,5P_3_-binding PH domain, where it is activated by phosphorylation at different sites by PDK1 and the TORC2 complex [10, 11]. In our yeast engineered model, PM tethering of the p110α catalytic subunit is achieved by adding N-terminal myristoylation or C-terminal CAAX prenylation signals to this protein [4, 5]. Reconstitution of class IA PI3K activity by this means results in yeast growth inhibition as a consequence of the withdrawal of essential PM PtdIns4,5P_2_ pools [12], as well as in localization and enhanced phosphorylation of Akt1 at the PM due to the production of PtdIns3,4,5P_3_[6]. Remarkably, expression of neither naked (non PM-directed) p110α nor Akt1 alone in yeast leads to growth defect, whereas co-expression of both heterologous proteins inhibits yeast growth in an Akt catalytic activity-dependent manner as a consequence of the interference of active Akt with yeast TORC2 signaling [13]. Thus, even in the absence of the p85 regulatory subunit or artificial targeting to the yeast PM, p110α can produce enough PtdIns3,4,5P_3_ to efficiently relocate and activate Akt. However, unlike myr-p110α and p110α-CAAX, naked p110α is not able to eliminate enough PtdIns4,5P_2_ from the yeast PM to compromise cell viability [5, 7].

Recent evidence suggests that the yeast PM is organized in microdomains and that PtdIns4,5P_2_ plays a role in configuring them [14, 15]. In turn, microdomains such as the eisosome/MCC (Membrane Compartment containing Can1) are involved in the control of PtdIns4,5P_2_ levels [15-17]. Here, we have designed chimeras to fine-tune the localization of heterologous PI3K activity in the yeast PM to two specific membrane microdomains that are stable along the budding cycle: the septin collar at the bud neck and eisosomes/MCCs. Moreover, in order to determine PI3K activity *in vivo* at both single-cell and whole population levels, we have developed novel fluorescent protein-based reporters that accurately permit assessment of heterologous PI3K activity in the yeast model.

## RESULTS AND DISCUSSION

### Spatial targeting of PI3K activity to specific yeast PM microdomains

**Figure 1:**
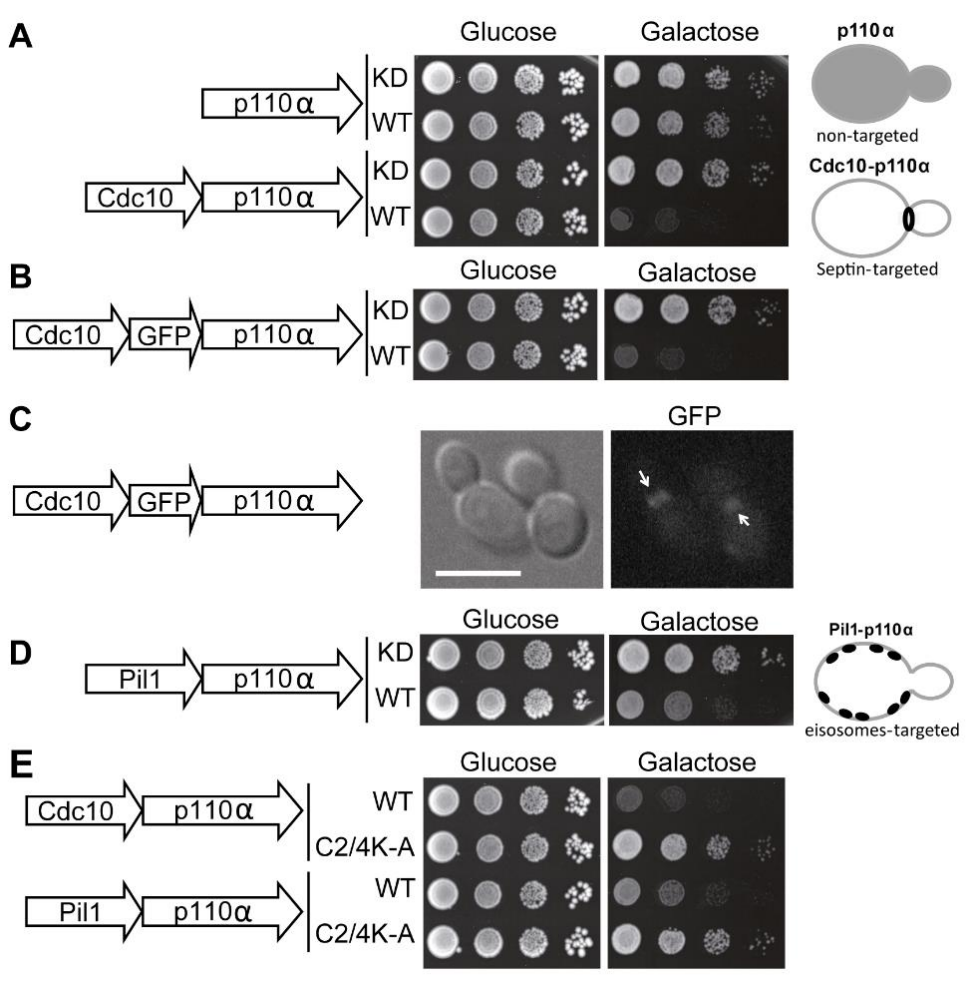
FIGURE 1: Effects on yeast growth of non-targeted and targeted p110α versions. **(A)** Ten-fold serial dilutions of wild type YPH499 cells bearing plasmids YCpLG-PI3Kα(K802R) [p110α(K802R)], YCpLG-PI3Kα (p110α), YCpLG-Cdc10-p110α(K802R) or YCpLG-Cdc10-p110α, respectively. Cells were cultured at 30°C on SD (Glucose) or SG (Galactose) agar for induction of p110α expression. Expressed fusions are indicated in the corresponding sketches at the left. **(B)** Yeast agar drop growth assays as in **(A)**, but of YCpLG-Cdc10-GFP-p110α(K802R) or YCpLG-Cdc10-GFP-p110α representative transformant clones. **(C)** Bright field and equivalent GFP fluorescence microscopy images of cells expressing the triple fusion protein [Cdc10-GFP-p110α-WT]. The arrows indicate localization at the septin ring at the mother-bud neck. The scale bar represents 5 µm. **(D)** Yeast growth assay of representative transformant clones bearing plasmids YCpLG-Pil1-p110α(K802R) or YCpLG-Pil1-p110α. **(E)** Growth of representative yeast clones expressing either wild type or C2-domain mutant (C2/4K-A) p110α versions of the Cdc10-p110α or Pil1-p110α fusion proteins, from plasmids YCpLG-Cdc10-p110α, YCpLG-Cdc10-p110α C2/4K-A, YCpLG-Pil1-p110α or YCpLG-Pil1-p110α C2/4K-A, as indicated.

N-terminal myristoylation or C-terminal prenylation signals, previously used to bring p110α in contact to its substrate [2, 4, 5], should indiscriminately direct PI3K activity to any location at the yeast PM. Instead, we decided to spatially modulate this activity to discrete domains in order to study the effects of locally enhanced conversion of PtdIns4,5P_2_ into PtdIns3,4,5P_3_. Through the budding cycle, the stretch of PM that conforms the bud neck is tightly and stably marked by a highly structured ring of septin filaments consisting of Cdc3, Cdc10, Shs1, Cdc11 and Cdc12 [18]. Septins are PtdIns4,5P_2_-binding proteins and the interaction with this phosphoinositide promotes their assembly into filaments [19]. Therefore, we decided to specifically direct PI3K activity to the septin-delimited PM area. To this end we produced a chimera consisting of the Cdc10 septin fused to the N-terminus of p110α. The Cdc10-p110α fusion protein resulted inhibitory for yeast cell growth and its toxicity relied on p110α catalytic activity, as a kinase-dead Cdc10-p110α(K802R) chimera was tolerated (Fig. 1A). To prove that Cdc10 in this sort of fusion was still tethered to the septin ring we developed a triple Cdc10-GFP-p110α chimeric protein. When expressed, this new chimera also inhibited yeast growth and specifically marked the bud neck (Fig. 1B-C). Furthermore, a point mutation in Cdc10 (Gly179 to Asp, known as the *cdc10-11* allele) which impairs its assembly at the ring [20, 21], led to loss of both localization (data not shown) and toxicity (Fig. S1A). These results implied that the toxicity caused by the septin-PI3K chimera relied on both the targeting to the septin ring, where the PI3K catalytic subunit should be close to its substrate at the PM, and on PtdIns4,5P_2_ consuming-PI3K activity. Despite the purported importance of PtdIns4,5P_2_ in septin assembly [19], Cdc10-p110α did not lead to phenotypes characteristic of loss of septin ring function [20], such as bud elongation (Fig. S1B). This observation hints that the PtdIns4,5P_2_ pool underlying septin filaments may not be accessible for PI3K or that Cdc10 and PI3K are competing for the binding to this pool. We next tested whether Cdc10, when fused to the catalytically inactive p110α mutant (K802R), whose expression does not affect yeast growth, was able to complement a loss-of-function *cdc10-11* mutant. The Cdc10-p110α(K802R) chimera was not able to support growth of this mutant at the restrictive temperature (Fig. S1C), suggesting that the presence of p110α bound to its C-terminus prevented Cdc10 from playing its functional role at the septin filaments in spite of its proper localization.

We then studied the influence of directing PI3K activity to the MCC. With this aim, we developed a second fusion by attaching to p110α the eisosome core component Pil1, an F-BAR domain-containing protein that binds curved furrow-like MCC microdomains [22]. As shown in Fig. 1D, tethering p110α to the eisosomes also led to growth inhibition, although to a lesser extent than that caused by Cdc10-p110α. Expression of Pil1-p110α did not interfere with eisosome assembly, since the localization of Sur7-GFP, a marker for these structures at the PM [23, 24], was apparently unaltered (Fig. S2A). Moreover, the kinase-dead Pil1-p110α(K802R) chimera was unable to complement alterations in eisosome assembly displayed by an *lsp1*∆ *pil1*∆ double mutant. As in the case of Cdc10-p110α(K802R), loss of Pil1 function in Pil1-p110α(K802R) must be a consequence of the bulky addition of p110 to its C-terminus, as restoration of a STOP codon (by site-directed mutagenesis in *PIL1* at the site of the fusion with the p110α cDNA in our expression plasmid) led to a functional Pil1 (Fig. S2B).

The p110α C2 domain is involved in PM recognition [25]. We previously showed that a quadruple mutation abolishing key positive charges within this domain (K410A, R412A, K413A, K416A), abrogated PI3K-dependent growth inhibition in our yeast model [5] when the interaction of p110α with the yeast PM was non-artificial, i.e., through recruitment by the regulatory subunit p85α. In contrast, the robust attachment of p110α by the CAAX motif made this construct insensitive to these multiple C2 mutations [5]. Both Cdc10 and Pil1 fusions to p110α seemed to mimic p85α function by bringing p110α in proximity to the PM, as the C2 domain of p110α was also necessary for the toxicity of these chimeras (Fig. 1E).

In sum, synthetically directing p110α to discrete scaffolds at the yeast submembrane, such as the septin ring or the eisosomes, simulates physiological recruitment by the PI3K regulatory subunit in higher cells without apparently interfering with the assembly and function of these structures, and leads to PtdIns4,5P_2_ depletion and toxicity due to PI3K activity.

### Differential effects on endocytosis of PI3K activity directed to septins vs. eisosomes

**Figure 2:**
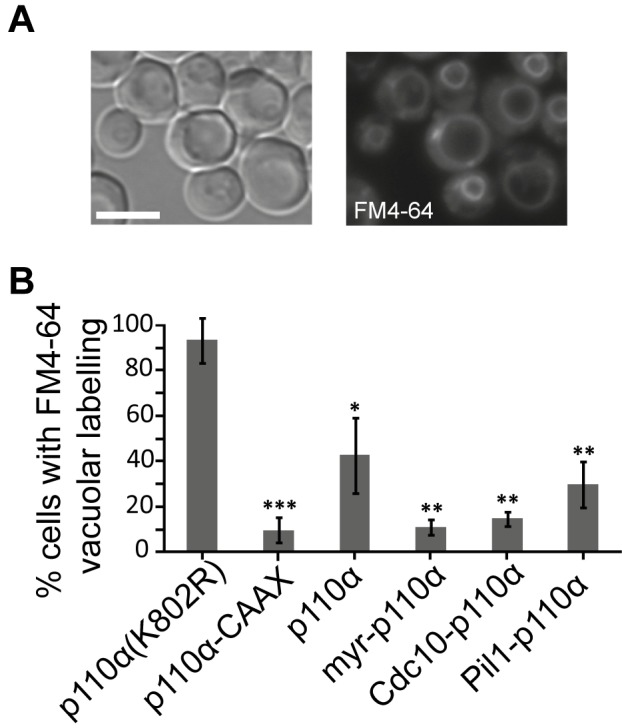
FIGURE 2: Endocytic fitness of non-targeted and various PM-targeted p110α versions. **(A)** Bright field (left image) or fluorescence microscopy (right image) representative images of typical FM4-64 vacuolar staining in YPH499 cells expressing the kinasedead mutant p110α(K802R), 60 min after the addition of the vital dye. **(B)** Graphical representation of the percentage of cells (n ≥ 300) showing the aforementioned vacuolar staining pattern when expressing p110α(K802R), p110α-CAAX, p110α, myr-p110α, Cdc10-p110α and Pil1-p110α. Results displayed in the graphs correspond to the mean of three biological replicates performed on different clones. Error bars represent the standard deviation (SD). Asterisks (*, **, ***) indicate a p-value < 0.05, < 0.01, < 0.001 respectively by the Student's t-test, referred to p110α(K802R). Scale bars indicate 5 µm.

Due to the essential role of PtdIns4,5P_2_ in endocytic pathways [26], expression of p110α-CAAX in yeast leads to an impairment of endocytosis, resulting in a defective internalization of the endocytic marker FM4-64 [12]. By monitoring the incorporation of this fluorescent dye into vacuolar membranes, the final destination of the endocytic pathway, we tested whether spatial direction of PI3K activity to either the septins or eisosomes would distinctly influence endocytosis, as compared to naked (non-membrane-directed), myristoylated (myr-p110α) or prenylated (p110α-CAAX) versions of p110α. All active p110α versions negatively affected endocytosis, being myr-p110α and Cdc10-p110α as efficient as p110α-CAAX (Fig. 2). Naked, non-PM-targeted p110α significantly decreased FM4-64 vacuolar labelling but was the less efficient version of p110α in doing so, as expected. Interestingly, Pil1-p110α was not as competent as Cdc10-p110α inhibiting endocytosis, in agreement with its lower toxicity, suggesting that tethering PI3K to the eisosomes had less impact in endocytosis than directing it to the bud neck. The role of eisosomes in endocytosis is controversial: while they were first described as sites of endocytosis [27], several reports favor the hypothesis that they may negatively regulate endocytosis by sequestering endocytic components [28]. In fact, they have been related to down-regulation of PtdIns4,5P_2_ levels via recruitment of the phosphoinositide-phosphatase Inp51/Sjl1 [17, 29]. Since Pil1-p110α, like Inp51/Sjl1, should locally deplete PtdIns4,5P_2_, it is conceivable that this fusion has less impact on endocytosis as compared to other PM-targeted p110α versions. In contrast, septins provide an important spatial landmark for the clustering of exo-endocytic machinery at the growing bud and at the septum [30], in consonance with a higher impact of PtdIns4,5P_2_ depletion on endocytosis from that spot.

### A tool for tracking PI3K activity in vivo at the single-cell level

**Figure 3:**
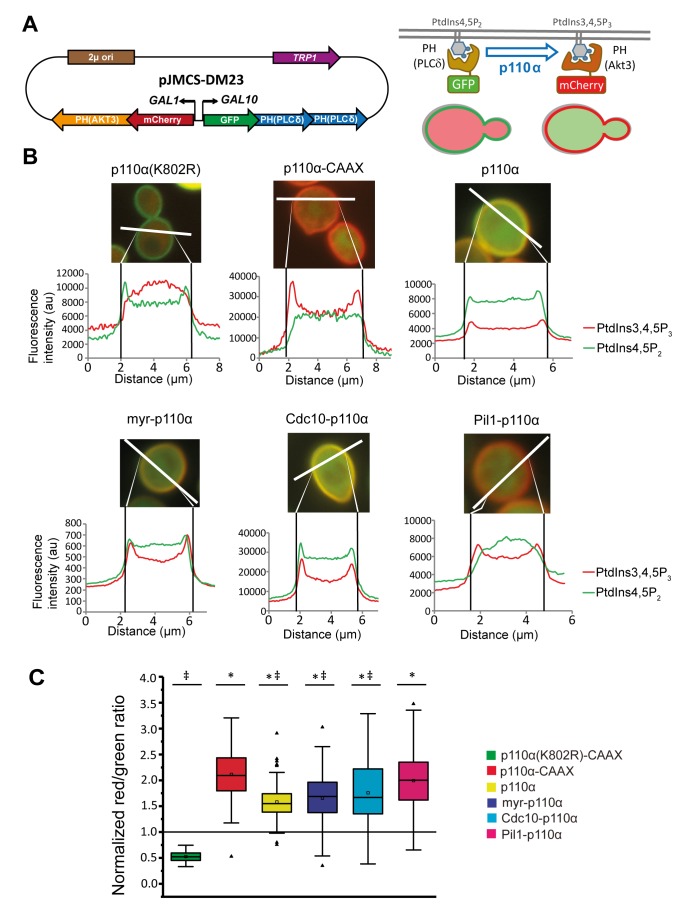
FIGURE 3: Dual Reporter for the Activity of PI3K (DRAPIK) strategy: a double marker to measure PtdIns4,5P_2_ and PtdIns3,4,5P_3_ at singlecell level. **(A)** Sketch of plasmid pJMCS-DM23, showing its main elements (left) and rationale and experimental design for the double marker analyses (right). Both fluorescent reporters are expressed simultaneously; yeast PM is rich in PtdIns4,5P_2_ and lacks PtdIns3,4,5P_3_, leading to recruitment of the green reporter to the PM whereas the red reporter remains cytoplasmic. Expression of PI3K should revert this situation proportionally to its enzymatic activity, the efficiency of its recruitment to the PM, and local substrate availability. **(B)** Representative fluorescence microscopy merged (red and green channels) images of YPH499 cells co-transformed with pJMCS-DM23 and expression plasmids for p110α(K802R), p110α-CAAX, p110α, myr-p110α, Cdc10-p110α and Pil1-p110α PI3K versions. Below, densitometric histograms showing the red (PtdIns3,4,5P_3_) and green (PtdIns4,5P_2_) fluorescence intensity in arbitrary units (au) along a transversal section of the cell, depicted above in white color, whose distance is represented in µm in abscissae. **(C)** Graph representing normalized red:green ratio [(PtdIns3,4,5P_3_) vs (PtdIns4,5P_2_)] for a population of individually monitored randomly chosen cells (n=90 per experiment) adjusted to a normal distribution. Cells co-express the same plasmids than in **(B)**. The symbols *, ‡ and # express statistical significance when compared to p110α-KD, p110α-CAAX or Pil1-p110α, respectively (Bonferroni p-value < 0.01 for two symbols and < 0.001 for three). The symbols ▫ and ― represent the average and median values of the population; the colored boxes cluster the data between 25% and 75% of the population; the bars with a capped end (I) cluster the data between 5% and 95% of the population; the outlier data are represented by the symbol ∆.

Once we had achieved subcellular targeting of heterologous p110α to discrete PM spots, we wondered whether this could lead to loss of particular PtdIns4,5P_2_ pools and spatially restricted generation of PtdIns3,4,5P_3_. In order to investigate this, we developed a double fluorescent reporter vector for tracking PI3K activity *in vivo* at the single-cell level, by using the bidirectional expression vector pESC-TRP1 as a platform. This vector bears the *GAL1-GAL10* co-inducible promoters facing opposing linkers. We fused two copies of the PH(PLCδ) PtdIns4,5P_2_-binding domain to GFP and the PH(Akt3) PtdIns3,4,5P_3_-binding domain to mCherry (Fig. 3A). In this fashion, the yeast PM would be stained in green in the absence of PI3K activity and the red marker would be recruited to the membrane only when PI3K was locally active. We named this experimental setting Dual Reporter for the Activity of PI3K (DRAPIK).

By fluorescence microscopy, we used DRAPIK to analyze the association of the green and red signals to the PM in yeast cells upon expression of various p110α versions. Representative cells are shown in Fig. 3B. As expected, cells bearing control kinase-dead p110α (K802R) had their PM marked only in the green channel, whereas p110α-CAAX fully reversed this situation and displayed a predominant peripheral red signal, suggesting that virtually all PtdIns4,5P_2_ was turned into PtdIns3,4,5P_3_, in line with the high toxicity in yeast of this version of PI3K [4]. Naked p110α led to detectable conversion of PtdIns4,5P_2_ into PtdIns3,4,5P_3_, congruently with its effect on endocytosis fitness shown above in Figure 2, and with its ability to activate its downstream kinase Akt through its PH domain, as previously reported [6, 13]. N-terminal myristoylation of p110α led to high red signal at the PM but it did not fully eliminate PtdIns4,5P_2_, as the green signal persisted, which explains why toxicity of myr-p110α in yeast is lower than that of p110α-CAAX [5] but higher than that of naked p110α. Peculiarly, direction of PI3K activity to either the septin ring or eisosomes did not lead to obvious local patterns of green to red conversion at these sites by DRAPIK analysis, but rather gave also rise to general change of PtdIns4,5P_2_ into PtdIns3,4,5P_3_ along the whole PM. This likely denotes that phosphoinositides can readily diffuse in the PM even if their modifying enzymes are immobilized in particular spots.

By analyzing individually a significant number of randomly chosen cells per experiment (n = 90), normalizing PM vs. cytoplasmic fluorescence signals for each channel and calculating red:green ratios, we got average data for each p110α version (Fig. 3C). Despite cell-to-cell variability, all p110α versions except the kinase-dead control had red:green average ratios > 1, the highest corresponding to p110α-CAAX and the lowest to naked p110α. These quantitative data are in line with our results above, indicating a correlation between PI3K-driven yeast growth inhibition and endocytosis defects, with the intriguing exception of the eisosome- and septin-directed chimeras. Pil1-p110α produced a very efficient conversion of PtdIns4,5P_2_ to PtdIns3,4,5P_3_, similar to that of p110α-CAAX, whereas the septin-PI3K fusion did not eliminate all PtdIns4,5P_2_. This suggests that there is a different requirement for PtdIns4,5P_2_-dependent functions in the environment of each of the PM subdomains studied. Eisosomes are ubiquitous spots distributed around the whole PM. Thus, it is reasonable that Pil1-p110α leads to a higher red signal throughout the whole PM since PtdIns4,5P_2_-PtdIns3,4,5P_3_ conversion is likely taking place all over the yeast PM. In spite of this, peculiarly, as discussed above, septin-directed PI3K activity, had a higher impact in endocytosis and viability than eisosome-directed PI3K.

### A tool for the study of PI3K activity in yeast cell populations that can be used to test PI3K inhibitors

**Figure 4:**
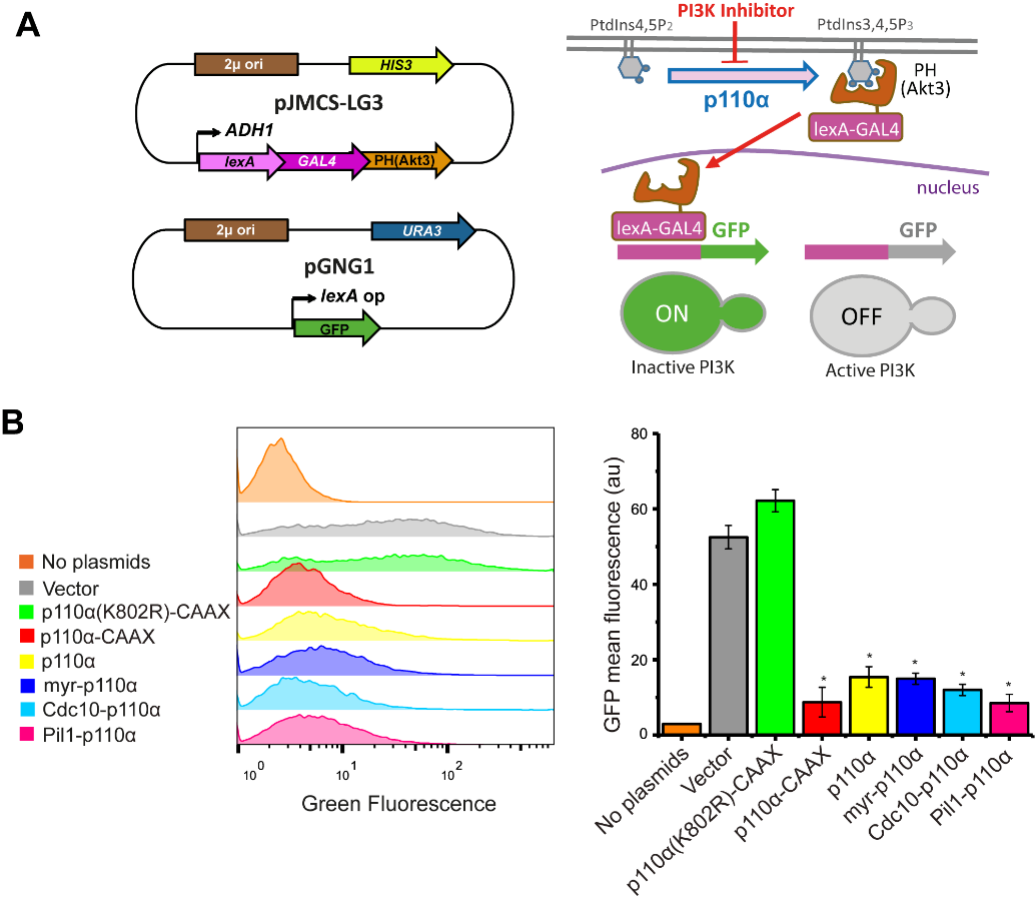
FIGURE 4: The FLUorescence by PI3K Inhibition (FLUPI) assay for the detection of heterologous PI3K activity by flow cytometry. **(A)** Sketches of plasmid pJMCS-LG3 bearing the transcriptional activator construct and pGNG1 (Mobitec) (left) and rationale of the system (right): the LexA-Gal4-PH(Akt3) reporter is retained at the PM when the PI3K product PtdIns3,4,5P_3_ is present. Its release due to low PI3K activity or its inhibition should proportionally lead to nuclear translocation of the chimeric transcription factor and GFP expression. **(B)** On the left, stacked histograms (n = 10,000) showing green fluorescence in abscissae of untransformed YPH499 strain (No plasmids) and triple co-transformant clones bearing pJMCS-LG3, pGNG1 and either empty YCpLG (Vector) or expressing one of the PI3K versions: p110α(K802R)-CAAX, p110α-CAAX, p110α, myr-p110α, Cdc10-p110α or Pil1-p110α. On the right, graph showing each population's mean GFP fluorescence (au) corresponding to the average of three biological replicates (n = 30,000). The asterisks (*) indicate a Bonferroni p-value < 0.001 vs. Vector; error bars represent SD.

The simultaneous double phosphoinositide marker described above allows single-cell analyses but was not amenable for quantitative analyses in large cell populations. Thus, we designed a fluorescence-based strategy that would readily allow monitoring PI3K activity in yeast cultures by flow cytometry. We fused an artificial LexA-Gal4 chimeric transcriptional activator to the PtdIns3,4,5P_3_- binding PH domain of Akt3. In a strain co-transformed with a second plasmid bearing a transcriptional LexA-dependent GFP reporter and a third plasmid expressing p110α, the LexA-Gal4-PH(Akt3) transcriptional activator would be sequestered at the PM by PI3K-generated PtdIns3,4,5P_3_ pool, precluding GFP expression. In contrast, yeast cells lacking PI3K activity would not retain the LexA-Gal4-PH(Akt3) fusion protein at the PM, allowing nuclear translocation and GFP expression (Fig. 4A). Since the system should lead to a gain of fluorescence upon PI3K inhibition, we named this experimental setting FLUorescence by PI3K Inhibition (FLUPI) assay. To test the system, we used as a proof-of-principle our collection of p110α versions. All active PI3K forms, regardless of the presence or absence of PM-directing signals, turned off GFP expression, showing a high sensitivity for detecting the presence of PtdIns3,4,5P_3_ at the PM and, therefore, PI3K activity (Fig. 4B). This assay confirmed that Pil1-p110α was as efficient as p110α-CAAX in producing PtdIns3,4,5P_3_ in the yeast cells, in spite of its lower toxicity.

**Figure 5:**
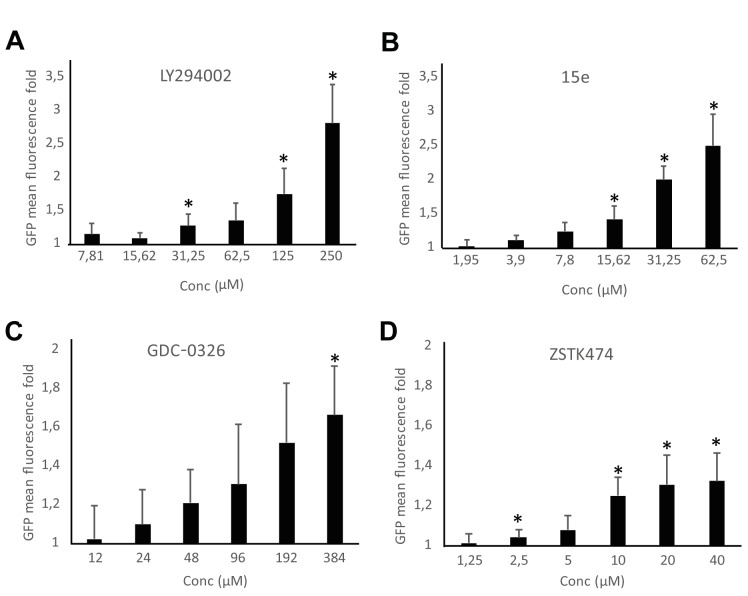
FIGURE 5: Dose-response graphs showing enhancement of fluorescence by the FLUPI assay. Triple pJMCS-LG3, pGNG1 and YCpLG-p110α YPH499 transformants were analyzed by flow cytometry as in Fig. 4B in the presence of different doses of inhibitors as noted. The abscissae represent fold increase in green fluorescence of treated samples with the indicated concentrations of compounds LY294002 **(A)**, 15e **(B)**, GDC-0326 **(C)** and ZSTK474 **(D)** relative to their correspondent DMSO alone control (normalized to 1). Three different clones were analyzed per experiment (n = 10,000 cells per clone). Data correspond to the average, and error bars represent SD. Asterisks (*) mark data points that are statistically significant respective to the data of the lowest concentration of compound tested in each series, according to Student's t-test (p < 0.05).

Bioassays for PI3K inhibition are useful in drug discovery. We previously reported a growth-based assay using as a platform our humanized yeast system [3]. However, fluorescence-based assays are more sensitive and versatile. The above-described DRAPIK system could be useful to trace PI3K inhibition at a single-cell level but, as it requires microscopy, it would not be easily scalable to high throughput screening unless high content analysis platforms were used. On the contrary, the FLUPI assay described here might be optimum for this purpose. Since unmodified p110α led to significant GFP fluorescence shutting off, we considered that this version of p110α should be more suitable for a simple PI3K inhibition assay as compared to the artificially PM-targeted p110α versions tested above. Thus, we tested in a FLUPI assay based on bare p110α a series of available chemical inhibitors for their ability to recover GFP fluorescence, namely LY294002 [31], the isoform-specific p110α inhibitors 15e [32] and GDC-0326, a benzoxazepin derivative [33], and the isoform-nonspecific triazine derivative ZSTK474 [34]. For all four compounds, a dose-dependent enhancement of fluorescence was observed in the range of concentrations tested (Fig. 5). As compared to the previously reported growth recovery assay on yeast expressing p110α-CAAX [3], in which we had also tested the LY294002, 15e and ZSTK474 inhibitors, the FLUPI assay also proved sensitive in the µM range for these compounds. Like in the previous setting, 15e was the most potent, leading to significant fluorescence increase at lower concentrations, whereas LY294002 required higher concentrations to reach the same effect. Fluorescence increase for GDC-0326 and ZSTK474 was more modest, but the latter was more efficient at low concentrations, similar to its performance in the growth recovery assay [3]. These data demonstrate the efficiency of the FLUPI bioassay to potentially screen for PI3K inhibitors on unmodified p110α using fluorescence-based strategies, suitable for microfluidics or other high-throughput platforms.

In sum, we have designed a toolkit for studying class IA PI3K in a versatile humanized yeast model that permits (i) directing PI3K activity to discrete localizations at the PM, (ii) monitoring its activity at the single-cell and population levels, and (iii) studying its pharmacological inhibition *in vivo*. These experimental settings broaden the experimental possibilities offered by the yeast cellular model on molecular and pharmacological studies based on this key oncoprotein.

## MATERIALS AND METHODS

### Strains, culture media and growth conditions

Yeast strains used in this work are listed in Table S1. YPD [1% (w/v) yeast extract, 2% (w/v) peptone and 2% (w/v) glucose] broth or agar was used for yeast growth as a general non-selective medium. Synthetic dextrose (SD) medium consisted of 0.17% yeast nitrogen base without amino acids, 0.5% ammonium sulfate, 2% glucose and 0.12% synthetic complete mixture drop out mix lacking the appropriate amino acids and nucleic acid bases to maintain plasmid selection. In SG (synthetic galactose) and SR (synthetic raffinose) media, glucose was respectively replaced with 2% (w/v) galactose or 1.5% (w/v) raffinose. For *GAL1* promoter induction, cells were cultured in SR broth for 18 h at 30°C, then the appropriate amount of these cultures was suspended into fresh SG to reach an OD_600_ of 0.3, and they were incubated for additional 4 - 6 h. Spot growth assays on agar were performed as described [4].

### Plasmids

Transformation of *E. coli* and yeast and other basic molecular biology methods were carried out using standard methods. All plasmids and oligonucleotides used are listed in Tables S2 and S3, respectively. *CDC10* and *CDC10-GFP* genes were amplified by regular PCR from pLA10 plasmid [20] using Cdc10-FW, Cdc10-Rv and Cdc10-GFP-Rv primers. *PIL1* was amplified from *S. cerevisiae* genomic DNA (BY4741) with Pil1-Fw and Pil1-Rv primers. A Gly-Ala (5×) linker was added to the reverse primers to facilitate protein folding. All primers were flanked by *Bam*HI sites and the resulting inserts were then digested with this enzyme and subsequently cloned in frame in plasmid YCpLG-PI3Kα yielding YCpLG-Cdc10-p110α, YCpLG-Cdc10-GFP-p110α and YCpLG-Pil1-p110α.

*Dpn*I-based site directed mutagenesis was performed using the QuikChange kit (Agilent). To generate *cdc10-11* allele-containing p110α fusions, the *cdc10(G179D)* mutation was introduced with primers Cdc10-G179D-Fw and Cdc10-G179D-Rv. The YCpLG-Pil1-STOP plasmid was obtained by introducing a stop codon in the *Bam*HI restriction site just before the *PIK3CA* gene coding sequence in YCpLG-Pil1-p110α, using the primers STOP-p110α-Fw and STOP-p110α-Rv. p110α(K802R) kinase-dead mutations and the p110α C2-domain mutant C2/4K-A (K410A, R412A, K413A, K416A) were obtained with the primers previously described [5].

DRAPIK plasmid: Double marker plasmid pJMCS-DM23 was constructed by molecular cloning in the yeast expression plasmid pESC-TRP (Agilent). First, GFPx2PH(PLCδ) was amplified by PCR with primers GFP-Fw and PH(PLCδ)-Rv using plasmid pRS426-GFP2XPH(PLCδ) [35] as template. Then, it was cloned in pESC-TRP by using *Spe*I*-Bgl*II restriction sites, yielding the plasmid pJMCS-DM20. The PH domain of Akt3 (residues 1-164) was amplified by PCR from pYES2-mCherry-Akt3 [13] with primers Cherry-Fw and PH(Akt3)-Rv and finally cloned into *Bam*HI*-Nhe*I sites of pJMCS-DM20 to generate pJMCS-DM23.

FLUPI plasmid: To construct the pJMCS-LG3 plasmid expressing a LexA-Gal4-PH(Akt3) fusion, a *Not*I restriction site was artificially inserted through site-directed mutagenesis with Insert-NotI-Fw and Insert-NotI-Rv primers into the pEG202-GAL4 plasmid (MoBiTec, Germany) disrupting the stop codon of *GAL4*. Then the PH(Akt3) domain was amplified from pJMCS-DM23 using *Not*I-PH(Akt3)-Fw and *Not*I-PH(Akt3)- RV primers and cloned into *Not*I sites of modified pEG202-GAL4, to generate pJMCS-LG3.

### Microscopy techniques, image processing and statistical analysis

For *in vivo* fluorescence microscopy (GFP or mCherry), cultures subjected to *GAL1* induction were concentrated by centrifugation at 3000 rpm for 2 min and visualized in an Eclipse TE2000U Nikon microscope. Digital images were captured with an Orca C4742-95-12ER camera and processed with HCI-mage software (Hamamatsu, Hamamatsu, Japan). Endocytosis dynamics was monitored by staining with FM4-64 (Fischer Scientific) as previously reported [36].

For DRAPIK assays, fluorescence microscopy images of cells co-transformed with pJMCS-DM23 and various YCpLG-p110α versions were analyzed as follows: measurements of both red (PtdIns3,4,5P_3_) and green (PtdIns4,5P_2_) fluorescence intensity through a line across one single cell were taken with FiJi software [37]. These values were displayed as single-cell densitometric histograms. As a quantitative approach, the “normalized red:green ratio” was calculated by measuring the fluorescence signal for both red and green channels with HCImage in two points at opposite sides of the PM and in one in cytoplasm. Then, for each channel, each PM fluorescence signal was normalized against the corresponding cytoplasmic fluorescence signal from the same cell to generate two PM:cytoplasm ratios per cell. The average between them becomes the final ”normalized red:green ratio” for each cell. Thirty randomly chosen cells were analyzed for each culture and triplicates of three different clones were analyzed. Thus, a total of 90 cells were analyzed per p110α version assayed. A two-way ANOVA using Bonferroni test was performed with Origin Pro statistics software (OriginLab Corp., Northampton, MA, USA).

### Flow cytometry

For FLUPI experiments, YPH499 yeast cells bearing pJMCS-LG3, pGNG1 and various YCpLG-p110α versions were cultured as for fluorescence microscopy analysis. For GFP expression analyses, cells were collected after 5 hours of galactose induction, fixed with 4% formaldehyde in PBS, washed with PBS and then analyzed by flow cytometry in Guava easyCyte or CELLQuest 3.3 (Becton Dickinson) flow cytometers, acquiring green fluorescence through a 488 nm excitation laser and a 525/30 BP emission filter (BFP). Data were processed using FlowJo software (FlowJo LLC, Ashland, OR, USA). In order to calculate statistical significance, a two-way ANOVA using Bonferroni test was performed with Origin Pro statistics software (OriginLab, Northampton, MA, USA).

For p110α inhibition assays, two-fold serial dilutions of 15e (”PI3Kα inhibitor II”, Echelon Biosciences, Salt Lake City, UT, USA), LY294002 (Echelon), GDC-0326 (Selleckchem, Houston, TX, USA) and ZSTK474 (Selleckchem), were performed in SG media. All the compounds were previously dissolved in dimethylsulfoxide (DMSO, Sigma-Aldrich) at stock concentrations of 32 mM, 29 mM, 100 mM and 50 mM, respectively. A negative control containing the maximum concentration of DMSO used in each assay was included. 200 µL of the yeast preinocula adjusted to an OD_600_ of 1 were added to every tube before incubation in a shaker at 30°C for 5 h. Inhibition assays were carried out as biological triplicates. Cells were collected and processed as mentioned above. At least 10,000 cells were analysed for each experiment. A green fluorescence ratio was obtained by dividing each raw value of mean GFP fluorescence at every concentration by the raw value of the DMSO control for each clone. Statistical significance was calculated by Student's t-test.

## SUPPLEMENTAL MATERIAL

Click here for supplemental data file.

All supplemental data for this article are also available online at http://microbialcell.com/researcharticles/a-humanized-yeast-based-toolkit-for-monitoring-phosphatidylinositol-3-kinase-activity-at-both-single-cell-and-population-levels/.

## Abbreviations:

DRAPIK: - Dual Reporter for the Activity of PI3K,
FLUPI: - FLUorescence by PI3K Inhibition,
MCC: - membrane compartment containing Can1,
PM: - plasma membrane.
