# Supplementary material for: A humanized yeast-based toolkit for monitoring phosphatidylinositol 3-kinase activity at both single cell and population levels
Source: Microb Cell. 2018 Nov 12;5(12):545–54. doi: 10.15698/mic2018.12.660 (PMC6282018; doi:10.15698/mic2018.12.660)
Supplement: Supplementary file 1 [file mic-05-545-s01.pdf]

A humanized yeast-based toolkit for monitoring phosphatidylinositol 3-kinase activity at both single cell and population levels

Julia María Coronas-Serna, Teresa Fernández-Acero, María Molina and Víctor J. Cid

SUPPLEMENTARY MATERIAL

SUPPLEMENTARY TABLES

Table S1. Yeast strains used in this work.

Strain name	Genotype	From
BY4741	*MATa; his3Δ1; leu2Δ0; met15Δ0; ura3Δ0*	EUROSCARF WGD collection
YPH499	*MATa; ade2-10; trp1-63; leu2-1; ura3-52; his3-Δ20; lys2-801*	[1]
1784	*MATα ura3-52 leu 2-3,112 trp1-1 his4 CanR*	[2]
VCY1	Isogenic to 1784, *cdc10-11*	[3]
VMY8	Isogenic to BY4741, *SUR7-GFP::URA3*	[4]
VMY9	*MATα lsp1Δ::kanMX4 pil1Δ::kanMX4 ura3Δ0 his3Δ1 leu2Δ0 SUR7-GFP::URA3*	[4]

Table S2. Plasmids generated and used in this work

Plasmid	Description	Source/Refererence
YCpLG	*CEN*, *LEU2*,*GAL1* promoter, empty vector.	[5]
YCpLG-PI3Kα	Expresses wild type p110α cDNA from the *GAL1* promoter	[6]
YCpLG-PI3Kα-CAAX	Expresses p110α-CAAX from the *GAL1* promoter	[6]
YCpLG-Myr-PI3Kα	Expresses Myr-p110α from the *GAL1* promoter	[6]
YCpLG-PI3Kα(K802R)	Expresses kinase-dead p110α(K802R) from the *GAL1* promoter	[6]
pLA10	Expresses Cdc10-GFP from its own *CDC10* promoter	[3]
YCpLG-Cdc10-p110α	Expresses Cdc10-p110α from the *GAL1* promoter	This work
YCpLG-Cdc10-GFP-p110α	Expresses Cdc10-GFP-p110α from the *GAL1* promoter	This work
YCpLG-Cdc10-p110α(K802R)	Expresses Cdc10-p110α(K802R) from the *GAL1* promoter	This work
YCpLG-Cdc10-GFP-p110α(K802R)	Expresses Cdc10-GFP-p110α(K802R) from the *GAL1* promoter	This work
YCpLG-*cdc10-11*-p110α	Expresses Cdc10(G179D)-p110α from the *GAL1* promoter	This work
YCpLG-*cdc10-11*-p110α(K802R)	Expresses Cdc10(G179D)-p110α(K802R) from the *GAL1* promoter	This work
YCpLG-*cdc10-11*-GFP-p110α	Expresses Cdc10(G179D)-GFP-p110α from the *GAL1* promoter	This work

YCpLG-*cdc10-11*-GFP-p110α(K802R)	Expresses Cdc10(G179D)-GFP-p110α(K802R) from the *GAL1* promoter	This work
YCpLG-Cdc10-p110α-(C2/4KA)	Expresses Cdc10-p110α-(C2/4KA) from the *GAL1* promoter	This work
YCpLG-Pil1-p110α	Expresses Pil1-p110α from the *GAL1* promoter	This work
YCpLG-Pil1-p110α(K802R)	Expresses Pil1-p110α(K802R) from the *GAL1* promoter	This work
YCpLG-Pil1-STOP	Pil1-STOP-p110α. Expresses Pil1 from the *GAL1* promoter	This work
YCpLG-Pil1-p110α(C2/4KA)	Expresses Pil1-p110α-(C2/4KA) from the *GAL1* promoter	This work
pESC-TRP	*2μ*, *TRP1*, *GAL1/GAL10* bidirectional promoter, empty vector.	Agilent Technologies
pRS426-GFP2XPH(PLCδ)	*2μ, URA3*, GFP-2×PH(PLCδ) fluorescent reporter for PtdIns4,5P_2	[7]
mCherry-Akt3-pYES3	Expresses mCherry-Akt3 from the *GAL1* promoter	I. Rodríguez-Escudero & Teresa Fernández-Acero, to be published elsewhere
pJMCS-DM23	GFP-2×PH (PLCδ)-mCherryPH(Akt3) double marker in pESC-TRP	This work
pJMCS-DM20	GFP-2×PH (PLCδ) in pESC-TRP	This work
pGNG1	GFP expression reporter Grow N'Glow™ system	MoBiTec
pEG202-GAL4	LexA-GAL4 positive control fusion for two-hybrid system	MoBiTec
pJMCS-LG3	Expresses the LexA-GAL4-PH(Akt3) fusion	This work

Table S3. Oligonucleotides used in this work.

Name	Sequence
Cdc10-Fw	(5'-CGGGATCCATGGACCCTCTCAGCTCAGTACAGC-3')
Cdc10-Rv	(5'-CGGGATCCGGCACCGGCTCCAGCGCCTGCACCAGCTCCACGTTGAATGGCGTTGCTAG-3')
Cdc10-GFP-Rv	(5'-CGGGATCCGGCACCGGCTCCAGCGCCTGCACCAGCTCCTTTGTATAGTTCATCCATGCC-3')
Pil1-Fw	(5'-CGGGATCCATGCACAGAACTTACTCTTTA-3')
Pil1-Rv	(5'-CGGGATCCGGCACCGGCTCCAGCGCCTGCACCAGCTCCAGCTGTTGTTTGTTGGGGA-3')
Cdc10-G179D-Fw	(5'-CAAATGTTATACCAGTTATTGACAAGTCGGATACATTGACTTTA-3')
Cdc10-G179D-Rv	(5'-TAAAGTCAATGTATCCGACTTGTCAATAACTGGTATAACATTTG-3').
STOP-p110α-Fw	(5'-AGCCGGTGCCTGATCCGCCACC-3')
STOP-p110α-Rv	(5'-GTGGCGGATCAGGCACCGGCTC-3')
GFP-Fw	(5'-GACTAGTATGGGTAAAGGTGTAGAAC-3')
PH(PLCδ) Rv	(5'-GAAGATCTCCCCTCGAGGTCGACTA-3')
Cherry-Fw	(5'-CGGGATCCATGGTGAGCAAGGGCGAG-3')
PH (Akt3)-Rv	(5'-CTAGCTAGCTTACAAAATAACTTTCCCAAAAG-3')
Insert-NotI-Fw	(5'-AAAGAGAGCGGCCGCTAAAATGAATCGTAGATACTGAAAAACCCCG-3')
Insert-NotI-Rv	(5'-ATTTTAGCGGCCGCTCTCTTTTTTTGGGTTTGGTGGGGTATCTTCA-3')
NotI-PH(Akt3)-Fw	(5'-AAGGAAAAAAGCGGCCGCATGAGCGATGTTACCATTGTG-3')
NotI-PH(Akt3)-RV	(5'-AAGGAAAAAAGCGGCCGCTTACAAAATAACTTTCCCAAAAGT-3')

SUPPLEMENTARY FIGURES

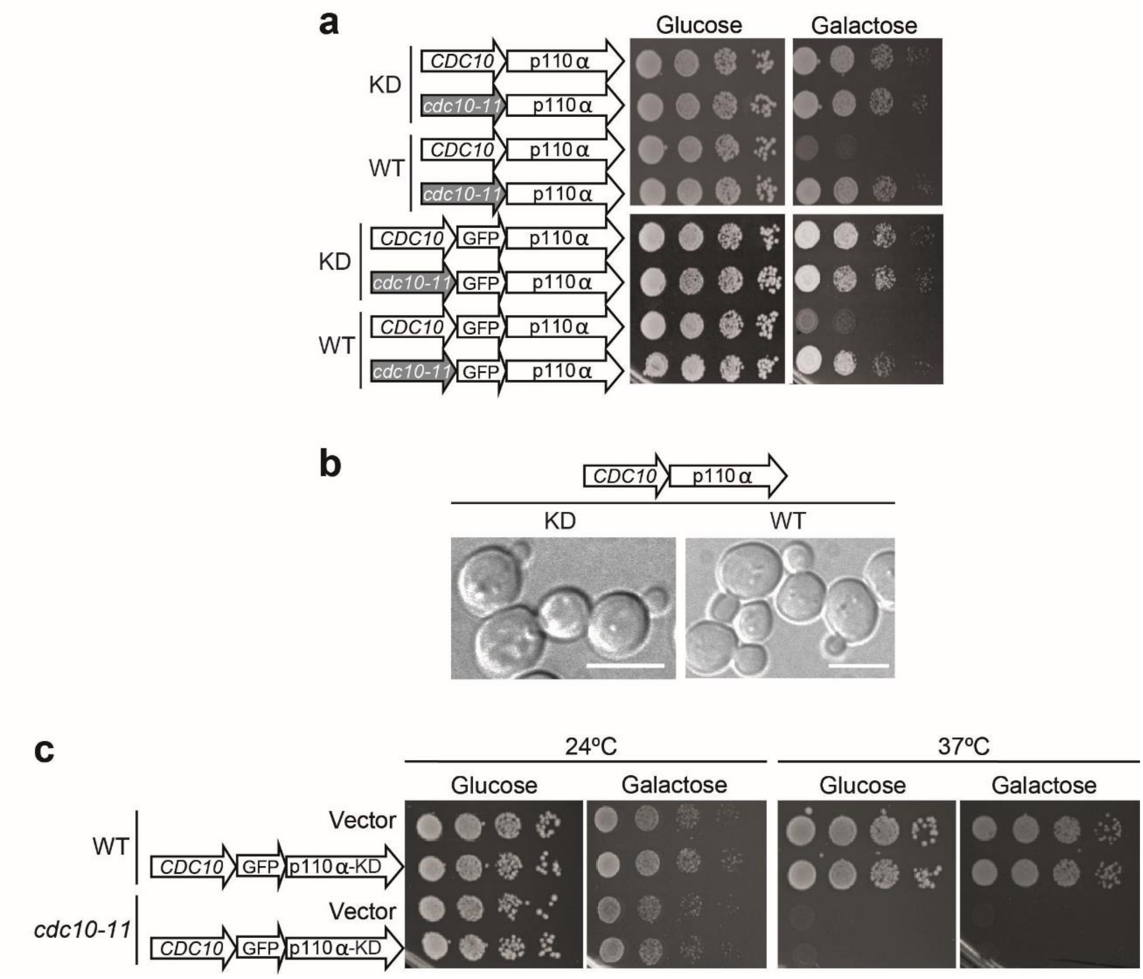

Figure S1. (a) Ten-fold serial dilutions of YPH499 yeast cells transformants bearing the plasmids YCpLG-Cdc10-p110α(K802R), YCpLG-*cdc10-11*-p110α(K802R), YCpLG-Cdc10-p110α, YCpLG-*cdc10-11*-p110α, YCpLG-Cdc10-GFP-p110α(K802R), YCpLG-*cdc10-11*-GFP-p110α(K802R), YCpLG-Cdc10-GFP-p110α, YCpLG-*cdc10-11*-GFP-p110α and cultured at 24°C on SD (Glucose) or SG (Galactose) agar plates. The expressed constructs are indicated on the sketches at the left. (b) Differential Interference Contrast (DIC) microscopy images of YPH499 cells expressing kinase-dead Cdc10-p110α(K802R) (KD, left) or wild type Cdc10-p110α (WT, right). (c) Ten-fold serial dilutions of wild type 1784 and the isogenic *cdc10-11* mutant VCY1 transformed with either empty vector (YCpLG) or the YCpLG-Cdc10-p110α(K802R) plasmid cultured in SD (Glucose) or SG (Galactose) at the indicated temperatures.

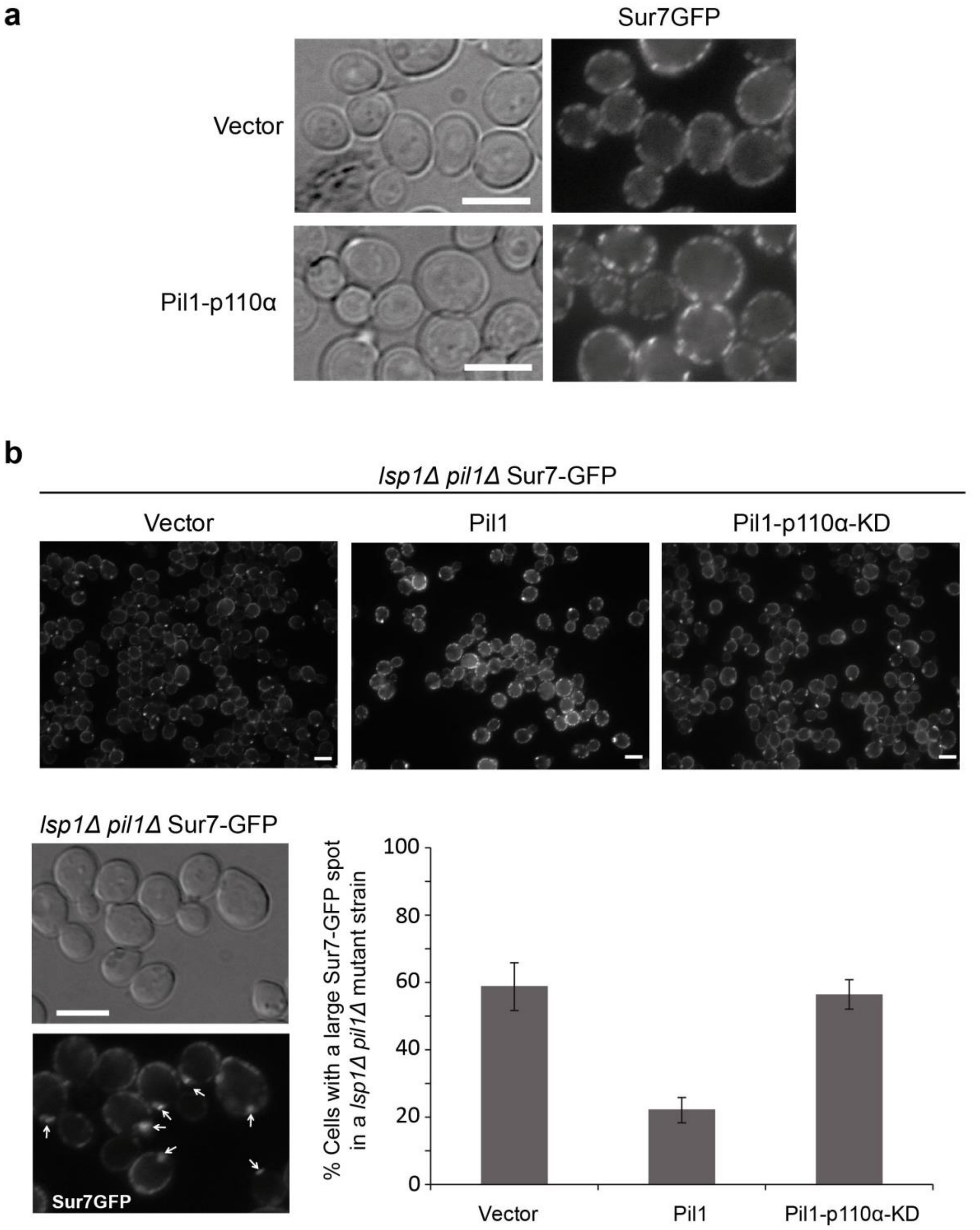

Figure S2. (a) Bright field and the corresponding GFP fluorescence microscopy images of representative cells of a Sur7-GFP tagged strain (VMY8), transformed with the empty vector YCpLG as a control or the plasmid YCpLG-Pil1-p110α. (b) On the upper side, GFP fluorescence microscopy images of a representative field of a *lsp1*Δ *pil1*Δ Sur7-GFP strain (VMY9) transformed with the empty vector YCpLG (Vector) or plasmids YCpLG-Pil1-STOP (Pil1) and YCpLG-Pil1-p110α(K802R) (Pil1-p110α-KD). On the lower side, at the left, representative field showing VMY9 cells typically displaying a large Sur7-GFP spot, marked by arrows. At the right side, a graph indicating the percentage of cells (n=30) with such spot in cultures expressing the same plasmids than in (a). The results correspond to three biological replicates and error bars represent SD.

SUPPLEMENTARY MATERIAL BIBLIOGRAPHY

1. Sikorski RS, and Hieter P (**1989**). A system of shuttle vectors and yeast host strains designed for efficient manipulation of DNA in Saccharomyces cerevisiae. **Genetics** 122(1): 19-27.
2. Lee KS, Irie K, Gotoh Y, Watanabe Y, Araki H, Nishida E, Matsumoto K, and Levin DE (**1993**). A yeast mitogen-activated protein kinase homolog (Mpk1p) mediates signalling by protein kinase C. **Mol Cell Biol** 13(5): 3067-3075.
3. Cid VJ, Adamikova L, Cenamor R, Molina M, Sánchez M, and Nombela C (**1998**). Cell integrity and morphogenesis in a budding yeast septin mutant. **Microbiology** 144(12): 3463-3474. doi: 10.1099/00221287-144-12-3463.
4. Mascaraque V, Hernaez ML, Jiménez-Sánchez M, Hansen R, Gil C, Martín H, Cid VJ, and Molina M (**2013**). Phosphoproteomic analysis of protein kinase C signaling in *Saccharomyces cerevisiae* reveals Slt2 mitogen-activated protein kinase (MAPK)-dependent phosphorylation of eisosome core components. **Mol Cell Proteomics** 12(3): 557-574. doi: 10.1074/mcp.M112.020438.
5. Bardwell L, Cook JG, Zhu-Shimoni JX, Voora D, and Thorner J (**1998**). Differential regulation of transcription: repression by unactivated mitogen-activated protein kinase Kss1 requires the Dig1 and Dig2 proteins. **Proc Natl Acad Sci U S A** 95(26): 15400-15405.
6. Rodríguez-Escudero I, Roelants FM, Thorner J, Nombela C, Molina M, and Cid VJ (**2005**). Reconstitution of the mammalian PI3K/PTEN/Akt pathway in yeast. **Biochem J** 390(2): 613-623. doi: 10.1042/bj20050574.
7. Stefan CJ, Audhya A, and Emr SD (**2002**). The yeast synaptojanin-like proteins control the cellular distribution of phosphatidylinositol (4,5)-bisphosphate. **Mol Biol Cell** 13(2): 542-557. doi: 10.1091/mbc.01-10-0476.
